# Evaluation of the Performance of an Indirect Immunofluorescence Assay for the Detection of Anti-MDA5 Antibodies

**DOI:** 10.3390/biomedicines10112969

**Published:** 2022-11-18

**Authors:** Anaïs Nombel, Jean-Jacques Pin, Nicole Fabien, Pierre Miossec, Frédéric Coutant

**Affiliations:** 1 Immunology Department, Lyon-Sud Hospital, Hospices Civils de Lyon, 69495 Pierre-Bénite, France; 2 Eurobio Scientific Dendritics—Edouard Herriot Hospital, 69003 Lyon, France; 3 Immunogenomics and Inflammation Research Team, University of Lyon, Edouard Herriot Hospital, 69003 Lyon, France; 4 Department of Immunology and Rheumatology, Edouard Herriot Hospital, 69003 Lyon, France

**Keywords:** myositis, dermatomyositis, idiopathic inflammatory myopathies, autoantibody, MDA5, indirect immunofluorescence, biomarker, diagnosis, immunomonitoring, follow-up

## Abstract

Anti-melanoma differentiation-associated protein 5 (MDA5) antibody (Ab) positive dermatomyositis (anti-MDA5 DM) is a rare systemic autoimmune disease; further, its prognosis can be rapidly fatal due to pulmonary involvement. The identification and quantification of anti-MDA5 Abs, which serve as a highly specific biomarker of the disease, is a critical step for the establishing of both the diagnosis and monitoring of the disease’s activity. The development of a simple, fast, low-cost, and specific detection system of anti-MDA5 Ab is therefore highly desirable for the purposes of routine laboratory diagnosis. Here, we developed a human cell line that stably expresses MDA5 and evaluated its analytical performance in order to detect anti-MDA5 Abs by the utilization of indirect immunofluorescence (IIF). Serum samples from 23 anti-MDA5 DM patients and 22 anti-MDA5 Abs negative myositis readings, which were obtained at time of diagnosis, were analyzed by IIF on MDA5-transfected cells. The results were compared with those obtained with specific semi-quantitative (immunodot) and quantitative (ELISA) assays. A specific cytoplasmic pattern was found solely with the sera of anti-MDA5 DM patients. The sensitivity and specificity of IIF on MDA5-transfected cells were 96% and 100%, respectively, compared with ELISA. The anti-MDA5 Abs titers that were determined by this approach were consistent with the quantitative results obtained by ELISA. Baseline concentrations of anti-MDA5 Abs, either by ELISA or IIF, were not significantly different between surviving and deceased patients; further, they did not differ significantly according to clinical phenotypes. Overall, an IIF cell-based assay constitutes a simple, fast, and low-cost approach to identify and quantify anti-MDA5 Abs; moreover, it is as efficient as ELISA.

## 1. Introduction

Dermatomyositis that is associated with anti-melanoma differentiation-associated protein 5 (MDA5) antibodies (anti-MDA5 DM) is a rare systemic autoimmune disease associated with severe symptoms, such as life-threatening rapidly progressive interstitial lung disease (RP-ILD) [1,2]. The clinical spectrum of anti-MDA5 DM can be divided into three clinical subgroups according to the predominance of cutaneous, articular, vascular, and pulmonary symptoms [3,4]. The mortality rate of this disease is the highest in the months following the diagnosis, whereas disease progression appears to settle down after this stage [5]. Combined immunosuppressive therapy initiated as soon as possible is considered to be the optimal approach in managing patients, which requires that the diagnosis be made as early as possible [6]. Although the knowledge on the pathogenic mechanisms of anti-MDA5 DM remains limited, the disease is characterized by an immune dysregulation. This is evidenced by the high production of type I interferon (IFN) and IFN-γ, the presence of antibodies (Abs) with immunostimulatory properties, as well as Abs that target a cytoplasmic viral sensor, i.e., the MDA5 protein [7,8,9]. 

Anti-MDA5 Abs constitute highly specific biomarkers of the disease and are essential, along with clinical features, for the diagnosis. The presence of anti-MDA5 Abs in the serum of patients can be detected via the utilization of indirect immunofluorescence (IIF) on HEp-2 cells, which is a widely used test in clinical practice for the detection of autoAbs [2]. Positive sera for anti-MDA5 Abs give rise to well-defined patterns on HEp-2 cells, which corresponds to a cytoplasmic staining in rare clustered cells and is associated with a higher risk in developing interstitial lung disease [9]. Specific immunoassays must always be conducted in order to confirm the identification of anti-MDA5 Abs, since the fluorescence pattern on HEp-2 cells is not consistently observed. To date, immunoprecipitation (IP) is considered to be the specific gold standard for detecting anti-MDA5 Abs [1]. However, IP is difficult to use in everyday practice as it is time-consuming, expensive, and difficult to interpret as several antigens targeted by myositis associated Abs comigrate with MDA5 [10,11,12]. For these reasons, the majority of medical laboratories have now opted for alternative immunoassays. Commercialized immunodot assays and enzyme-linked immunosorbent assays (ELISA) have been developed and validated with a reported specificity of, respectively 96–99% and 98%. When compared with the IP assay, there is a sensitivity of 75–93% and 100%, respectively [13,14,15]. ELISA allows the quantification of anti-MDA5 Abs, while immunodot assays only give a semi-quantitative result and are sometimes difficult to interpret in the lower intensities, especially when the clinical presentation is not typical. Accurate quantification or, at least, estimation of the titer of anti-MDA5 Abs in serum samples from patients is of relevance due to the fact that some studies have suggested that anti-MDA5 Ab levels correlate with clinical severity and response to treatment, which, therefore, may predict disease outcome [15,16,17,18,19,20]. However, the anti-MDA5 Abs predictive prognostic value at the admission stage is not clearly established and there are diverging results depending on studies that are consulted [20,21,22,23]. 

We developed a semi-quantitative IIF cell-based assay that allows expression in the cell of high levels of natively folded human MDA5 proteins. Its analytical performances were compared to ELISA by using sampled sera from a cohort of patients with anti-MDA5 DM, as well as patients with anti-MDA5 Abs negative myositis as the control group. We also determined, in our cohort, whether the baseline anti-MDA5 Abs titers could predict disease outcome and if higher titers were associated with more severe symptoms, which has been described by others [15,16,17,18,19,20].

We found that use of IIF on MDA5-transfected cells was efficient when detecting serum anti-MDA5 Abs that are easily identified by a cytoplasmic staining. Anti-MDA5 Abs titers determined by this approach were consistent with the quantitative results obtained by ELISA. However, neither ELISA concentrations, nor IIF titers predicted disease outcome. 

## 2. Materials and Methods

### 2.1. Patients and Sera

Sera were obtained from 23 adult patients sampled at the admission to the Hospices Civils de Lyon (France) and diagnosed with anti-MDA5 DM between January 2012 and December 2021. The diagnoses were made according to biological criteria (i.e., the presence of anti-MDA5 Abs in the serum) and clinical features: a DM skin rash compatible with DM according to the European NeuroMusclar Center (ENMC) criteria or Sontheimer criteria [24,25]; cutaneous symptoms compatible with DM (Gottron’s papules, palmar papules, and ulcerations); myositis; arthralgia; or ILD without other etiology. Clinical data for each patient and the control group were collected from the medical record. The following features were reported: ILD; RP-ILD; cutaneous symptoms; ulcerations; Raynaud phenomenon; arthralgia/arthritis and muscular disease (proximal muscles weakness, elevated CPKs, or pathologic features showing the presence of inflammatory infiltrates on biopsies when performed); as well as prognosis. 

Blood samples were collected before the initiation of the treatment. The identification of anti-MDA5 Abs in the sera was performed by immunodot assay, which is the routine technique used in our laboratory. From 2012 to 2021, the first technique used in the laboratory was the dot-blot (D-tek, BlueDiver, Mons, Belgium), which was quickly replaced by the line-blot technique (Line blot LIA, Euroimmun, Lübeck, Germany). Among the sera from patients with anti-MDA5 DM, the presence of anti-MDA5 Abs was determined with a line blot for 22 sera and a dot blot for one serum. Twenty-two sera from age- and gender-matched patients with anti-MDA5 Ab negative myositis were used as controls. Positivity for other Abs was also reported when available (Table 1).

The study (595-SCMDA5) was approved by the institutional review board for ethics (i.e., the Ethics Committee of the Hospitals of Lyon for the protection of people—Number 1 October 2022). The study was conducted in accordance with the Declaration of Helsinki ethical principles, as well as good clinical practices. 

### 2.2. Immunodot Assays

Anti-MDA5 Abs were assessed with commercialized immunodot assays (Line blot LIA, Euroimmun, Lübeck, Germany and/or dot blot D-Tek, BlueDiver, Mons, Belgium) according to the manufacturer’s instructions. Briefly, multiple purified antigens targeted by myositis-specific Abs (MSA) are fixed on the test strips, which are then incubated with the sera. MSA present in the sera form a complex with their specific antigen. Alkaline phosphatase conjugated anti-human IgG Ab is used as a secondary Ab. Alkaline phosphatase catalyzes a color reaction after the addition of its substrate (nitro blue tetrazolium chloride/5-bromo-4-chloro-3-indolyl phosphate), thereby producing a dark line at the antigen position. The intensity of the staining is quantified via the EUROLineScan software (Lubeck, Germany), which provides a semi-quantitative value of the Ab concentration. According to the manufacturer’s instructions, a sample is considered positive when the scanned intensity of the signal is greater than 5 for dot blots (dot D-tek) or 15 for line blots (dot Euroimmun).

### 2.3. Enzyme-Linked Immunosorbent Assay

Sera were stored at −80 °C until they were assessed by ELISA according to the manufacturer’s instructions (anti-MDA5 ELISA Kit, Medical & Biological Laboratories, Nagoya, Japan). Serum samples were diluted at 1:101. Assay diluent was used as a calibrator 1 (0 U/mL) and anti-MDA5 Abs diluted in assay diluent were used as calibrator 2 (100 U/mL). The low control was the human serum in assay diluent and high control was contained in the anti-MDA5 Ab in assay diluent. Horseradish peroxidase conjugated goat anti-human IgG Ab was used as a secondary Ab. Absorbance of each well was read at 450 nm and anti-MDA5 Ab levels were calculated following the manufacturer’s instructions: Unit value (U/mL) = 100 × (A450<Sample>–A450<Calibrator1>)/(A450<Calibrator 2>–A450<Calibrator 1>). The cut-off level was 32 U/mL, as previously determined [15].

### 2.4. Generation of a Stable Cell Line Expressing MDA5

The stable cell line expressing MDA5 was obtained by transfecting HEK293 cells with the plasmid pUNO harboring full-length MDA5 (Invivogen, Toulouse, France). Monolayer of HEK293 cells (200 × 10^3^ cells) were transfected by mixing 1 µg of plasmid with 1 µL polyethylenimine (~Mw 25k, Sigma-Aldrich, St. Louis, MO, USA), then diluted in 50 µL of DMEM (Eurobio scientific, Les Ulis, France). The stably transfected HEK293 cells were selected and maintained in DMEM supplemented with 10% FBS, glutamine, antibiotics (penicillin and streptomycin), and the selection antibiotic blasticidin (20 μg/mL, Invivogen). In order to enrich the cell culture with positive cells, several rounds of cloning by limit dilution were conducted until the generation of more than 90% of stable cells expressing MDA5. Cells stably expressing MDA5 were then mixed with untransfected HEK293 cells in a 1:5 ratio and cultured in 96-well plates (1 × 10^4^ cell/well) for 48 h at 37 °C in order to easily discriminate, via IIF, the specific staining from the nonspecific background in each well. 

### 2.5. Indirect Immunofluorescence on MDA5-Transfected HEK293 Cells

Serum samples were tested for anti-MDA5 Abs via IIF staining on the MDA5-transfected cells that were fixed with acetone in 96-wells plates, as previously described [26]. A fluorescein isothiocyanate (FITC)-conjugated goat anti-human IgG,A,M antibody (BI2114; Abliance, Compiègne, France) was diluted at 1:300 in PBS and used as a secondary Ab, which was contrary to the ELISA method that uses an anti-IgG only. The choice of using anti-IgG,A,M Abs, rather than anti-IgG Abs as secondary Abs in the IIF test, is justified by the fact that it has previously been reported that different isotypes of anti-MDA5 antibodies are detectable in the serum of patients, including the different subtypes of IgG, IgA, and IgM [27]. Moreover, the FITC anti-IgG,A,M Abs used in this study did not generate disturbing background noise for reading. Sera from patients and controls were diluted in PBS at 1:50, 1:100, 1:200, and 1:400. Positive samples were then further diluted at 1:450, 1:1350, 1:4050, and 1:12,150. Four known anti-MDA5 positive sera were pooled, diluted at 1:300 in PBS and used as a positive control. The pool of serum from healthy subjects was used as a negative control. Diluted serum (100 µL) was added to each well and incubated for 45 min at 37 °C. The wells were then washed twice with PBS. Diluted secondary Ab (50 µL) was added in each well and the plates were incubated for 1 h at 37 °C. Then, after another round of washing with PBS and a final wash with water, the plates were read using a fluorescence microscope (Zeiss Axioplan 2, Munich, Germany) by two independent readers.

### 2.6. Indirect Immunofluorescence on HEp-2 Cells

IIF was performed on HEp-2 cells (Bio-Rad laboratories, Marnes-la-Coquette, France). Sera were diluted at 1:160 and incubated on HEp2 cells for 30 min at room temperature. After three washes in phosphate-buffered saline (PBS), the slides were incubated with a FITC anti-human immunoglobulin (Ig)G (F(ab’)2) (diluted at 1:100) (Bio-Rad) for 30 min at room temperature. The slides were then read using a fluorescence microscope (Olympus, Hamburg, Germany).

### 2.7. Detection of Other Autoantibodies

An immunodot assay (Line blot LIA, Euroimmun, Lübeck, Germany and/or dot blot D-Tek, BlueDiver, Mons, Belgium) was used to identify anti-histidyl-tRNA synthetase (anti-Jo1) Abs, anti-isoleucyl-tRNA synthetase (anti-OJ) Abs, anti-Mi2 Abs and anti-tripartite motif containing 21 (TRIM21) Abs. Anti-cyclic citrullinated peptide (CCP) Abs, anti-Jo1 Abs, anti-centromere B (CENB) Abs, anti-small ribonucleoprotein (SmRNP) Abs, anti-Sjögren’s syndrome related antigen (SSA) Abs, and anti-TRIM21 Abs were all detected using a Luminex technology (Bioplex 2200, Biorad, Marnes la Coquette, France). Anti-3-hydroxy-3-methylglutaryl-CoA reductase (HMGCR) Abs were identified using a chemiluminescent immunoassay (QUANTA Flash^®^ HMGCR, Werfen, France). Finally, IIF staining on polynuclear cells allowed the detection of anti-neutrophil cytoplasm Abs (ANCANOVALite^®^ ANCA, Werfen, France). 

### 2.8. Statistical Analysis

Statistical comparisons were performed using the Student’s t-test, the Wilcoxon–Mann–Whitney U test, and Fisher’s exact test. All statistical analyses were carried out using RStudio software Version 2021.09.2., *p*-values < 0.05 were considered significant. 

## 3. Results

### 3.1. Comparison of Baseline Features and Outcomes of Anti-MDA5 Positive and Anti-MDA5 Negative DM Patients

Twenty-three patients diagnosed with anti-MDA5 DM (MDA5^+^ group) and 22 patients diagnosed with anti-MDA5-negative myositis (MDA5^−^ group) were enrolled in this study. The control group (MDA5^−^ group) was composed of 20 patients with anti-synthetase syndrome (18 possessed a positive serum for anti-Jo1 Abs, whereas 2 had anti-OJ Abs positivity). Further, there were 2 patients with immune necrotizing myopathy associated with anti-HMGCR Abs. The mean age at diagnosis in the MDA5^+^ group was 53.6 (range 22–80) years and 52.6 (23–77) years in the MDA5^−^ group (ns). Thirteen (57%) of anti-MDA5 DM patients were women. There was no significant difference between the two groups with respect to sex (ns). Anti-MDA5 DM patients had the highest prevalence of RP-ILD (43% vs. 0%, *p* < 0.001) and cutaneous manifestations (91% vs. 36%, *p* < 0.001), including ulcerations (30% vs. 0%, *p* < 0.01). Contrary to what is frequently found in the literature [1,5,10,18,21,28], the MDA5^+^ group was not associated with a lower frequency of muscle damage (65 vs. 59%, ns). The occurrence of arthritis and Raynaud phenomenon was comparable between the two groups (74 vs. 50%, ns and 22 vs. 18%, ns, respectively). The prognosis of patients with anti-MDA5^+^ DM was less favorable as seven patients died in this group, whereas one death was reported in the MDA5^−^ group (*p* < 0.05). Regarding serological features, anti-TRIM21 (also known as anti-SSA52) Abs were found in four patients of the MDA5^+^ group and six patients of the MDA5^−^ group. Among the MDA5^+^ group, one patient was positive for anti-Mi2 Abs, one for anti-CCP Abs, one for anti-SmRNP Abs, and one for anti-SSA60 Abs. One patient from the MDA5^−^ group had anti-centromere B Abs and one other was positive for ANCA (Table 1). 

**Table 1:** Clinical and serological features of anti-MDA5 positive and anti-MDA5 negative DM patients.

	Anti-MDA5^+^ DM (n = 23)	Anti-MDA5^–^ Myositis (n = 22)	*p*-Value
**Mean age ± SD (years)**	54 ± 15	53 ± 15	ns
**Women**	13/23 (57%)	17/22 (77%)	ns
**ILD**	18/23 (78%)	15/22 (69%)	ns
**RP-ILD**	10/23 (43%)	0 (0)	<0.001
**Cutaneous symptoms**	21/23 (91%)	8/22 (36%)	<0.001
**Skin ulcerations**	7/23 (30%)	0 (0)	<0.01
**Muscular manifestations**	15/23 (65%)	13/22 (59%)	ns
**Arthritis**	17/23 (74%)	11/22 (50%)	ns
**Raynaud phenomenon**	5/23 (22%)	4/22 (18%)	ns
**Mortality outcome**	7/23 (30%)	1/22 (5%)	<0.05
**Anti-MDA5 Ab**	23/23 (100%)	0 (0)	
**Anti-Jo1 Ab**	0 (0)	18/22 (82%)	
**Anti-OJ Ab**	0 (0)	2/22 (9%)	
**Anti-HMGCR Ab**	0 (0)	2/22 (9%)	
**Anti-Mi2 Ab**	1/23 (4%)	0 (0)	
**Other autoAb**	Anti-TRIM21 (4/23), anti-CCP (1/23), anti-SmRNP (1/23), anti-SSA60 (1/23)	Anti-TRIM21 (6/22), ANCA (1/18), anti-CENB (1/18)	

Abbreviations—ANCA: anti-neutrophil cytoplasm antibodies, CENB: centromere B, CCP: cyclic citrullinated peptide, DM: dermatomyositis, HMGCR: 3-hydroxy-3-methylglutaryl-CoA reductase, ILD: interstitial lung disease, Jo1: anti-histidyl-tRNA synthetase antibody, MDA5: melanoma differentiation-associated protein 5, ns: not significant, OJ: anti-isoleucyl-tRNA synthetase antibody, RNP: ribonucleoprotein, RP-ILD: rapidly progressive interstitial lung disease, SSA: Sjögren’s-syndrome-related antigen A, and TRIM21: tripartite motif containing-21.

### 3.2. Sensitivity and Specificity of the Anti-MDA5 Ab Cell-Based Specific Assay

**Figure 1:**
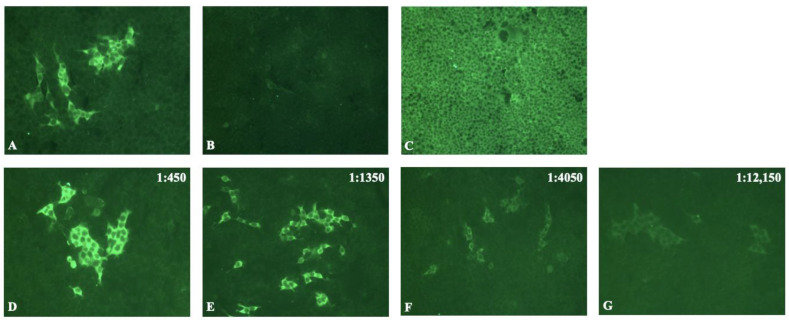
Indirect immunofluorescence on MDA5-transfected cells. Cells stably expressing MDA5 (>90% of transfected cells) were mixed with untransfected HEK293 cells in a 1:5 ratio and cultured in 96-well plates in order to easily discriminate by IIF the specific staining from the nonspecific background in the wells. A FITC anti-human IgG,A,M antibody (1:300) was used as a secondary antibody. (**A**) IIF obtained with the pooled sera of four patients with anti-MDA5 DM (positive control diluted at 1:50). (**B**) Pooled sera of healthy subjects (negative control diluted at 1:50). (**C**) Serum from one patient with anti-Jo1 Abs with a diffuse nonspecific staining in all cells. (**D**–**G**) Serum from one patient with anti-MDA5 antibodies that possessed positive DM diluted at 1:450 (**D**), 1:1350 (**E**), 1:4050 (**F**), and 1:12,150 (**G**), which was still slightly positive at the last dilution.

To easily discriminate, via IIF, the specific staining of anti-MDA5 Abs from the nonspecific background in the same well, HEK293 cells stably expressing MDA5 (>90% of transfected cells) were mixed with untransfected cells in a 1:5 ratio and cultured for 6 h in order to allow the cells adhere to the 96-well plates. MDA5-transfected cells were fixed with acetone. Further, the pooled sera of healthy subjects or the group of anti-MDA5 DM were used as negative and positive controls, respectively. As expected, anti-MDA5 Abs provided a specific IIF cytoplasmic staining on fixed MDA5-transfected cells, in contrast to the pooled sera of healthy subjects (Figure 1A,B). The tested sera from the MDA5^−^ group were negative in the majority of cases, or they could give rise to non-specific staining that was easily distinguishable from the specific staining, as illustrated by the intense cytoplasmic staining in all the cells observed in the serum of a patient with anti-synthetase syndrome associated with anti-Jo1 Abs (Figure 1C). Sera were considered positive for anti-MDA5 Abs when at least one fluorescent cluster of cells was detected in a well. Serial dilutions ranging from 1:50 to 1:12,150 were performed with each serum in order to define the titer of anti-MDA5 Abs as the inverse last positive dilution (Figure 1D–G). 

In order to evaluate the efficiency of the IIF assay to detect anti-MDA5 Abs, we analyzed the sera of the MDA5^+^ and MDA5^−^ group by the uses of IIF and ELISA. All sera from the MDA5^+^ group were positive as determined via ELISA, with titers ranging from 66 to 181 U/mL. In addition, 83% of sera also showed a specific MDA5 pattern on HEp-2 cells (detailed in Supplemental Table S1). All sera from the MDA5^−^ group were negative as determined via ELISA. The concordance rate between the two IIF readers was 100%. A total of 22 sera out of 23 from the MDA5^+^ group gave a specific cytoplasmic staining with the IIF assay (sensitivity = 96%). One serum, which gave the lowest titer of anti-MDA5 Abs when determined via ELISA (66 U/mL versus mean 129 U/mL, Table S1), was negative when determined via IIF. This serum was associated with a nonspecific diffuse cytoplasmic IIF staining in all the cells, most likely due to the high level of autoAbs that were other than anti-MDA5 Abs (Figure S1). It is therefore likely that the presence of these autoAbs masked the specific staining by anti-MDA5 Abs that were quantitatively less abundant in this serum. None of the sera from the MDA5^−^ group gave a specific MDA5 staining on the MDA5-transfected cells (specificity = 100%). 

### 3.3. Correlation between the Anti-MDA5 Antibody Levels Measured by Specific IIF Assay and via ELISA

**Figure 2:**
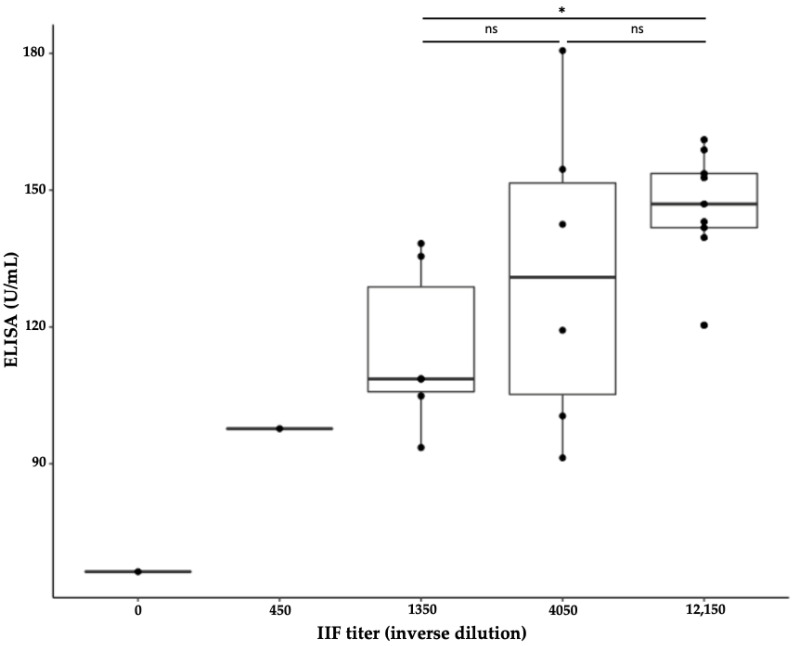
Anti-MDA5 antibody levels measured via specific IIF assay and via ELISA. ELISA titers were compared between three groups of patients as defined by their IIF titer (1350, 4050, and 12,150). Indirect immunofluorescence (IIF) titers that were negative or of 450 were excluded from the analysis due to the low number of samples. *: *p*-value < 0.05 and ns: not significant.

In order to evaluate the efficiency of the IIF-specific cell-based assay to quantify accurately anti-MDA5 Abs, sera quantifications obtained via IIF and via ELISA were compared. Patients were divided into three groups depending on their IIF titer (IIF 1350, IIF 4050, and IIF 12,150). Moreover, anti-MDA5 Ab quantifications were compared to those obtained via ELISA. The sera with a reading of IIF negative or positive when at a dilution lower than 1:1350 were too rare (only one serum in each condition) and were, therefore, excluded from this analysis. We found a significant increase in ELISA concentrations between the group IIF 1350 and IIF 12,150 (mean ELISA: 114 U/mL vs. 146 U/mL, *p* < 0.05) showing that in the IIF-specific cell-based assay, an increase or a decrease in two dilutions (i.e., a dilution factor of 3) was associated with a significant variation of anti-MDA5 Abs as measured via ELISA (Figure 2). 

### 3.4. Association of Anti-MDA5 Antibody Levels with Clinical Manifestations and Disease Severity

Several studies highlighted that anti-MDA5 Ab levels could be used to monitor the disease’s activity, especially the severity of ILD and cutaneous manifestations [15,16,17,18]. Furthermore, one study suggested that baseline levels of anti-MDA5 Abs could predict the outcome of patients, with lower levels of anti-MDA5 Abs observed in surviving patients when compared to deceased patients [16]. However, these results are conflicting [20,22,23]. In order to assess the prognostic value of anti-MDA5 levels at baseline in our cohort, we compared different features between the same three previous groups of patients depending on their IIF titer (comparison two-by-two: 1350 vs. 4050, 4050 vs. 12,150, and 1350 vs. 12,150) (see Table 2). First, we compared the prognosis of patients between those three groups and found no significant difference. Similarly, the mean ELISA values between surviving and deceased patients did not differ significantly (mean ELISA = 126 U/mL vs. 135 U/mL, ns). We next sought to compare the frequency of patients with ILD who had a rapidly progressive course between the three groups and found no significant difference. Similar results were observed in regard to the ELISA concentrations (mean ELISA = 133 U/mL in the group of patients with RP-ILD vs. 129 U/mL in the group with a chronic pulmonary damage or absence of pulmonary involvement, ns). We also did not identify any association between the levels of anti-MDA5 Abs and the age or sex of the patients, whatever the symptoms. 

In order to further study the possible association between the anti-MDA5 Ab levels at the baseline and the severity of all the symptoms associated with anti-MDA5 DM, we classified the anti-MDA5^+^ patients into three clinical subgroups, as previously described [3,22]. In this previous classification, the first group was mainly composed of women with RP-ILD and mechanic’s hands; furthermore, this group possessed the highest mortality rate (80%). The second group was made of men with skin vasculopathies (Raynaud phenomenon, skin ulcers, digital necrosis, and calcinosis), proximal muscle weakness, and fewer RP-ILD (23%); this group was of intermediate prognosis. Finally, the third group of women with arthralgia/arthritis and rare RP-ILD had the best prognosis. 

The patients of our cohort were then classified into three subgroups. This was achieved by taking into account the frequency of RP-ILD, cutaneous manifestations, ulcerations, Raynaud phenomenon, and arthritis (Supplemental Table S2). The first group was characterized by severe pulmonary damage (100% of RP-ILD) and included 11 patients who were mostly women (55%), with a mean age of 61 years and a poor prognosis (55% of mortality). The second group was composed of three patients with cutaneous vascular manifestations, such as ulcerations (100%) and Raynaud phenomenon (100%). No death was reported in this group. Finally, patients of the third group (n = 9) had frequent joint involvement (89%) and cutaneous symptoms (100%). Anti-MDA5 Ab levels measured by IIF and ELISA titers were compared between group 1 and group 3. Group 2 comprised too few patients to be included in the statistical analyses. We found no significant difference in the anti-MDA5 IIF titer between the two groups. Similarly, when studying anti-MDA5 Abs concentrations that were obtained by ELISA, the patients of group 1 did not have significantly higher anti-MDA5 Abs titers (135 U/mL vs. 129 U/mL, ns) (Table 3). 

## 4. Discussion

In this study, we developed and validated the efficiency of IIF on MDA5-transfected cells in a cohort of patients with known anti-MDA5 positive DM and patients with anti-MDA5 negative myositis. The IIF technique achieved an analytical sensitivity and specificity comparable to those of ELISA and immunodot assays, which validates its use for the detection of anti-MDA5 DM. The IIF assay also possessed certain valuable advantages over the gold standard immunoprecipitation technique. Firstly, all the laboratories specialized in the detection of autoAbs are familiar with the IIF technique. This IIF test, therefore, does not require any additional equipment. In addition, it does not involve the use of radioactive substances, which raises safety problems, and it does not require advanced technical training. It provides a result quickly (less than three hours to perform and read the test) and is easily interpretable, unlike the immunoprecipitation technique. Secondly, ELISA is another interesting technique that allows the quantification of anti-MDA5 Abs. However, the cost of the commercialized anti-MDA5 ELISA kit is about 4 to 5 times higher than the cost of the IIF assay, which may be prohibitive for routine use. A limitation of the IIF test is illustrated in our study with the serum containing the lowest concentration of anti-MDA5 Abs (66 U/mL in ELISA) and high levels of other autoAbs targeting cytoplasmic antigens that are other than the MDA5 protein. In this case, the specific staining associated with anti-MDA5 Abs is most likely masked by the presence of other autoAbs. The serum must then be analyzed using another specific immunoassay, such as ELISA or immunodot.

Previous studies reported a possible association between anti-MDA5 Ab levels and disease severity, outcomes, and response to treatment [15,16,17,18,19,20]. Interestingly, the presence of a cytoplasmic staining associated with the presence of anti-MDA5 Abs on HEp-2 cells was previously associated with a higher risk of developing ILD [9]. We showed that IIF on MDA5-transfected cells could be a valuable method used to quantify anti-MDA5 Abs as efficiently as ELISA. However, the analysis of the association between baseline levels of anti-MDA5 Abs (both in ELISA and IIF on transfected cells) and the outcome did not reveal statistical difference between surviving and deceased patients. This result is coherent with other reports that demonstrated that the baseline anti-MDA5 Ab levels did not differentiate between the patients who survived from those who succumbed to the disease [21,22]. Consistent with this, the levels of anti-MDA5 Abs also did not differ significantly between patients with or without RP-ILD, nor between the three clinical phenotypes associated with different outcomes. These results tend to support the notion that anti-mDA5 Ab levels are of limited value as short-term markers of disease activity but are useful markers for determining whether stable remission has been achieved, as well as for predicting relapse [20,22,23]. Increasing the number of patients in our cohort and the number of sampling time-points for each patient would allow us to confirm or disprove this notion. 

In conclusion, we developed a simple, fast, and cost-effective IIF assay on MDA5-transfected cells, which demonstrated a high concordance with a quantitative anti-MDA5 Abs via ELISA. It will be important to evaluate this assay on a larger cohort by including different time-points sampling in order to further explore the relevance of monitoring the titer of these autoantibodies to better predict stable remission or relapse.

## Figures and Tables

**Table 2:** Main severe features and demographic characteristics of patients depending on their anti-MDA5 Ab levels as obtained via indirect immunofluorescence conducted on transfected cells.

IIF Titer (Inverse Dilution) *	1350 (n = 6)	4050 (n = 6)	12150 (n = 9)	*p*-Value
**Mortality outcome**	2/6 (33%)	3/6 (50%)	2/9 (22%)	ns
**RP-ILD**	3/6 (50%)	3/6 (50%)	4/9 (44%)	ns
**Mean age ± SD (years)**	56 ± 7	57 ± 19	52 ± 18	ns
**Women**	3/6 (50%)	4/6 (67%)	4/9 (44%)	ns

* Patients with a negative or less than 1350 IIF titer were excluded (n = 2). Abbreviations—IIF: indirect immunofluorescence, RP-ILD: rapidly progressive interstitial lung disease, and ns: not significant.

**Table 3:** IIF and ELISA results of the three clinical subgroups of patients with anti-MDA5 Abs positive dermatomyositis.

	Group 1 (n = 11)	Group 2 (n = 3)	Group 3 (n = 9)	*p*-Value *
**IIF titer (inverse dilution)**				
**0**	0 (0)	1/3 (33%)	0 (0)	
**450**	0 (0)	1/3 (33%)	0 (0)	
**1350**	4/11 (36%)	0 (0)	2/9 (22%)	ns
**4050**	3/11 (28%)	0 (0)	3/9 (33%)	ns
**12,150**	4/11 (36%)	1/3 (33%)	4/9 (45%)	ns
**Mean ELISA ± SD (U/mL)**	135 ± 27	106 ± 44	129 ± 23	ns

* The *p*-values were obtained from Fisher’s test or Wilcoxon’s test between groups 1 and 3. Abbreviations—ELISA: enzyme linked immune sorbent assay, IIF: indirect immunofluorescence, and ns: not significant.

## Data Availability

The datasets generated for this study are available on request to the corresponding author.

## Supplementary Materials

The following supporting information can be downloaded at: https://www.mdpi.com/article/10.3390/biomedicines10112969/s1. Table S1: Results of immunodot assay, ELISA, and indirect immunofluorescence on MDA5-transfected cells for the 23 patients with anti-MDA5 positive dermatomyositis. Table S2: Clinical features of the three clinical subgroups defined on the frequency of RP-ILD, cutaneous manifestations, ulcerations, Raynaud phenomenon, and arthritis.
