# Supplementary material for: Evaluation of the Performance of an Indirect Immunofluorescence Assay for the Detection of Anti-MDA5 Antibodies
Source: Biomedicines. 2022 Nov 18;10(11):2969. doi: 10.3390/biomedicines10112969 (PMC9687661; doi:10.3390/biomedicines10112969)
Supplement: Supplementary file 1 [file biomedicines-10-02969-s001.zip › biomedicines-1979003-supplementary.pdf]

Supplementary materials

Table S1 : Results of immunodot assay, ELISA and indirect immunofluorescence on MDA5 transfected cells and HEp-2 cells for the 23 patients with anti-MDA5 positive dermatomyositis

Patient ID	Immunodot	Immunodot intensity	ELISA (U/mL)	IIF on MDA5 transfected cells (inverse dilution)	IIF pattern on HEp-2 cells
**MDA5^{+} 1**	positive	85	108	1350	MDA5 pattern
**MDA5^{+} 2**	positive	56	138	1350	MDA5 pattern
**MDA5^{+} 3**	Hpo	103	94	1350	Non-specific
**MDA5^{+} 4**	Hpo	114	143	12150	MDA5 pattern
**MDA5^{+} 5**	positive	85	119	4050	MDA5 pattern
**MDA5^{+} 6**	Hpo	103	105	1350	MDA5 pattern
**MDA5^{+} 7**	Hpo	112	181	4050	MDA5 pattern
**MDA5^{+} 8**	Hpo	179	153	12150	MDA5 pattern
**MDA5^{+} 9**	positive	46	91	4050	MDA5 pattern
**MDA5^{+} 10**	positive	44	159	12150	MDA5 pattern
**MDA5^{+} 11**	positive	31	100	4050	MDA5 pattern
**MDA5^{+} 12**	Hpo	175	142	12150	MDA5 pattern
**MDA5^{+} 13**	Hpo	140	147	12150	MDA5 pattern
**MDA5^{+} 14**	positive	39	109	1350	MDA5 pattern
**MDA5^{+} 15**	positive	76	135	1350	MDA5 pattern
**MDA5^{+} 16**	positive	38	155	4050	Non-specific
**MDA5^{+} 17**	positive	60	120	12150	MDA5 pattern
**MDA5^{+} 18**	Hpo	136	161	12150	MDA5 pattern
**MDA5^{+} 19**	positive	42	154	12150	Non-specific
**MDA5^{+} 20**	Hpo	127	140	12150	MDA5 pattern
**MDA5^{+} 21**	positive	72	98	450	MDA5 pattern
**MDA5^{+} 22**	positive	81	143	4050	Non-specific
**MDA5^{+} 23**	positive	74	66	0	MDA5 pattern

The IIF pattern on HEp-2 cells was termed "MDA5 pattern" when the characteristic cytoplasmic staining in rare clustered cells was observed. Otherwise, it was termed "Non-specific".
Abbreviations: Hpo: high positive, IIF: indirect immunofluorescence.

Table S2 : Clinical features of the three clinical subgroups made depending on the frequency of RP-ILD, cutaneous manifestations, ulcerations, Raynaud phenomenon and arthritis

	Group 1 (n=11)	Group 2 (n=3)	Group 3 (n=9)
**Mean age ± SD (years)**	61 ± 13	50 ± 13	46 ± 15
**Women**	6/11 (55%)	2/3 (67%)	5/9 (56%)
**Clinical manifestations**			
**RP-ILD**	11/11 (100%)	0 (0)	0 (0)
**Cutaneous manifestations**	9/11 (82%)	3/3 (100%)	9/9 (100%)
**Ulcerations**	4/11 (36%)	3/3 (100%)	0 (0)
**Arthritis**	6/11 (55%)	3/3 (100%)	8/9 (89%)
**Raynaud phenomenon**	2/11 (18%)	3/3 (100%)	0 (0)
**Muscular damage**	6/11 (55%)	3/3 (100%)	6/9 (67%)
**Mortality rate**	6/11 (55%)	0 (0)	1/9 (11%)

Abbreviations: RP-ILD: rapidly progressive interstitial lung disease
